# Impact of Metabolic Surgery on Gestational Diabetes Mellitus: A Cohort Analysis

**DOI:** 10.1111/ajo.70051

**Published:** 2025-05-28

**Authors:** Jasmine Wintour, Sarjana Afrin, Nicole Buxton, Mercy Madzivanyika, Katie Wynne

**Affiliations:** ^1^ Department of Diabetes and Endocrinology John Hunter Hospital Newcastle New South Wales Australia; ^2^ School of Medicine and Public Health University of Newcastle Newcastle New South Wales Australia; ^3^ Department of General Medicine John Hunter Hospital Newcastle New South Wales Australia; ^4^ Department of Nutrition and Dietetics John Hunter Hospital Newcastle New South Wales Australia; ^5^ Department of Obstetrics and Gynaecology John Hunter Hospital Newcastle New South Wales Australia; ^6^ Hunter Medical Research Institute ‘Equity in Health & Wellbeing’ Program Newcastle New South Wales Australia

**Keywords:** bariatric surgery, gestational diabetes mellitus, metabolic surgery, obesity, sleeve gastrectomy

## Abstract

**Background:**

With the upsurge of obesity in Australia and worldwide, the incidence of metabolic surgery is increasing in women of reproductive age. Metabolic surgery reduces the rate of gestational diabetes mellitus, however, the risk remains about that for the general population.

**Aim:**

To evaluate maternal and perinatal outcomes of consecutive women with GDM post‐metabolic surgery presenting to an endocrine antenatal clinic in a single tertiary centre.

**Methods:**

A retrospective cohort study of women with GDM after metabolic surgery were audited between 2020 and 2024.

**Results:**

This cohort of 36 women were aged 32.9 (± 4.7) years and 3.3 (±2.0) years post‐surgery with the majority undergoing sleeve gastrectomy (97.2%). Gestational diabetes was diagnosed at 25 (±6^+0^) weeks. Screening identified carbohydrate inadequacy or inconsistency (18/36, 50%) and micronutrient deficiency (34/36, 94.4%) were common, with 26/36 (72.2%) women deficient in more than one micronutrient. Insulin therapy was required in 16 women. Recurrent hypoglycaemia occurred in 10 (27.8%) women. Women birthed at 38 (±1^+0^) weeks, with unplanned Caesarean delivery in six (16.7%) women. Composite adverse neonatal outcomes occurred in 22/36 (61.1%) of births. Hypoglycaemia (< 2.6 mmol/L) occurred in 8/36 (22.2%) of neonates; 1/36 (2.8%) were small‐for‐gestational age, and 2/36 (5.6%) large‐for‐gestational age. A higher neonatal birthweight was observed in women with reported pre‐pregnancy BMI ≥ 30 kg/m^2^ and women requiring insulin.

**Conclusion:**

Nutritional deficiencies occur commonly in women with gestational diabetes after metabolic surgery. There was a high rate of composite adverse neonatal outcome suggesting that these pregnancies may be higher risk. Further research is required to evaluate the optimal methods of screening and recommended glycaemic targets.

## 1 Introduction

The global obesity crisis persists, with 1 in 8 people worldwide affected by obesity [1, 2]. Obesity is an independent risk factor for gestational diabetes (GDM) and several other complications, including, miscarriages, congenital anomalies, fetal macrosomia, Caesarean section deliveries, hypertensive disorders and admissions to neonatal intensive care units [3]. Although surgery reduces the rates of GDM compared to pre‐surgical weight; the risk of hypertensive disorders, macrosomia and GDM remain elevated above the general population [4, 5], with GDM conferring an increased risk of macrosomia and twice the rate of congenital malformations [6, 7].

The Australian National Health Survey in 2017–2018 reported 67% of adults were overweight or obese. The number of metabolic surgeries is increasing [8] as this is the most effective and permanent treatment [9]. Metabolic surgery is recommended for people with a body‐mass index (BMI) over 35 kg/m [2], regardless of the presence, absence, or severity of comorbidities and for patients with type 2 diabetes and BMI > 30 kg/m [2]; it should be considered in individuals with BMI over 30 kg/m^2^ who do not achieve substantial or durable weight loss or comorbidity improvement using nonsurgical methods [10]. Generally, weight loss of 20%–32% is achieved 1–2 years post‐surgery and is sustained over 20 years [11]. According to the Australian Bariatric Surgery Registry, over 15,000 surgeries were completed in 2023, with almost 80% female and over half were in the age range 20–44 years [12]. Of these, 30% plan pregnancy and it is likely their fertility will improve [13]. The obstetric management of women after metabolic surgery has become a significant clinical challenge for physicians and obstetricians. There is a critical need for preconception counselling to highlight the optimal timing of pregnancy, which should be delayed for 12–24 months or until weight loss plateaus and optimisation of nutrition has been achieved [14, 15]. To date, the majority of available literature focuses on patients following Roux‐en‐Y Gastric Bypass with more limited information available for women following sleeve gastrectomy. The popularity of sleeve gastrectomy continues to rise and now accounts for 80% of primary metabolic surgery procedure type [12].

Metabolic surgery may increase the risk of small‐for‐gestational‐age infants, nocturnal hypoglycaemia, shorter gestation without an increase in preterm birth [5], and nutritional deficiencies. The most common deficiencies after surgery are vitamin B12, vitamin D and other fat‐soluble vitamins, folate, calcium, iron and protein [14, 15] which, when severely deficient, can result in adverse pregnancy outcomes and congenital anomaly [13]. Compared to the standard nutritional recommendations for pregnancy, recommendations for daily dietary intake of nutrients are higher in pregnancies after metabolic surgery [15]. Sleeve gastrectomy is often associated with fewer nutritional deficiencies and surgical complications than Roux‐en‐Y gastric bypass surgery and is commonly the surgical procedure of choice in women of childbearing age [16].

The diagnosis of GDM in pregnancy after metabolic surgery is complicated by the changes in glucose homeostasis; the standard criteria for the oral glucose tolerance test (OGTT) are not evidence‐based in this population and the test may cause harm. Maternal plasma glucose concentrations after oral glucose load are characterised by a rapid glycaemic excursion and subsequent reactive hypoglycaemia: classically this glucose profile demonstrates hyperglycaemia at 1‐h post glucose administration then hypoglycaemia at 2‐h [4]. For example, 90% of women with a Roux‐en‐Y bypass experienced hypoglycaemia (< 2.8 mmol/L) following a 3‐h 75 g OGTT after at least 8 h of fasting [17]. The rates of hypoglycaemia vary according to the type of metabolic surgery: Roux‐en‐Y bypass (83.3%), sleeve gastrectomy (54.5%) and adjustable gastric band (11.8%) [17]. Notably, women with hypoglycaemia during a glucose challenge have a higher risk of repeated hypoglycaemia during pregnancy and consequent intrauterine growth restriction leading to lower birthweight neonates [18]. Throughout pregnancy, Roux‐en‐Y gastric bypass and sleeve gastrectomy may cause postprandial hyperinsulinaemic hypoglycaemia, nocturnal hypoglycaemia and wide glycaemic variability, partly due to an exaggerated release of glucose‐mediated incretins, e.g., glucagon‐like peptide 1 (GLP‐1) [4]. Importantly, as yet there is no evidence that treatment of post‐surgical patients with GDM diagnosed by an OGTT benefit from improved pregnancy outcomes [19].

## 2 Methods

This retrospective cohort study was conducted using data derived from consecutive women attending the antenatal endocrinology clinic at John Hunter Hospital between January 2020 and April 2024. Women were included if they were aged ≥ 18 years, had a previous history of metabolic surgery, and a diagnosis of GDM (at least one fasting glucose ≥ 5.1 mmol/L or at least two 2‐h post‐prandial glucose levels ≥ 6.7 mmol/L over a week of monitoring as per local protocol or if an OGTT had been performed any of a fasting plasma glucose ≥ 5.1 mmol/L, 1‐h plasma glucose ≥ 10.0 mmol/L, or 2‐h plasma glucose ≥ 8.5 mmol/L). Women were excluded if they had pre‐existing diabetes mellitus or if the majority of their antenatal care had occurred at a different site. Local guidelines recommend measurement of serum copper, selenium, zinc, international normalised ratio (INR as a surrogate for vitamin K), and vitamin B1, once during pregnancy; in addition to iron studies, vitamin D, vitamin B12 and serum folate levels each trimester. Maternal hypoglycaemia was defined as < 3.8 mmol/L and recurrent hypoglycaemia was defined as ≥ 2 occurrences. Adverse neonatal outcomes included neonatal hypoglycaemia defined as < 2.8 mmol/L, small‐for‐gestational age defined as < 10th centile, large‐for‐gestational age defined as > 90th centile, jaundice, neonatal intensive care admission, respiratory distress, resuscitation or shoulder dystocia and as a composite of these parameters. The study protocol was reviewed by the Hunter New England Ethics Committee and a waiver of consent was approved.

Data was analysed using GraphPad Software, Boston, USA and GIGAcalculator, Web Focus LLC, Sofia, Bulgaria. Descriptive statistics were presented as an absolute number ± standard deviation or percentage. Variables with normal distribution were presented as mean ± standard deviation (SD) while those that were not normal were presented as median ± interquartile range (IQR). Characteristics were compared using Student's *t*‐test for continuous variables and Chi‐square or Fisher's exact test for categorical variables. A *p* value < 0.05 was considered significant.

## 3 Results

There were 38 women who met the inclusion criteria. Two women were excluded; one with pre‐existing Type 2 diabetes and one who received their antenatal care at a different hospital. There were 36 patients included in the final cohort (Table 1) with mean age 32.9 (± 4.7) years, and 3 patients (8.3%) identified as Aboriginal or Torres Strait Islander, compared to the local population prevalence of 4.2%. The cohort pre‐pregnancy weight of 89.8 (± 24.5) kg was elevated compared to the National average of 72kg [20]. Metabolic surgery had occurred 3 [2, 4] years prior to conception; sleeve gastrectomy had been performed in 35/36 (97%) and the remaining participant had a gastric bypass. Of these women, 6/36 (16.7%) reported symptomatic hypoglycaemia prior to pregnancy. Obstetric booking appointment occurred at 16^+3^ (± 4^+5^).

**TABLE 1:** Baseline characteristics of the cohort of women with GDM after metabolic surgery (*n* = 36).

Characteristics	
Mean Age (SD), years	32.9 (4.7)
Aboriginal or Torres Strait Islander, *n* (%)	3 (8.3)
Smoking status, *n* (%)	5 (13.9)
Median gravidity (IQR)	3 (2, 4)
Median parity (IQR)	1 (0, 2)
Median pre‐pregnancy BMI (kg/m^2^), (IQR)	31.4 (28.3, 35.8)
< 25, *n* (%)	1 (2.8)
25–29.9, *n* (%)	14 (38.9)
≥ 30, *n* (%)	21 (58.3)
Metabolic surgery type	
Sleeve gastrectomy, *n* (%)	35 (97.2)
Gastric bypass, *n* (%)	1 (2.8)
Roux‐en‐Y gastric bypass (%)	0 (0)
Median time since surgery, years (IQR)	3 (2,4)
Median peak weight (*n* = 27), kg (IQR)	128 (113.5, 156)
Mean nadir weight (*n* = 20), kg (SD)	79.5 (21.9)
Mean total body weight percentage reduction from pre‐surgery weight to nadir weight (*n* = 20), %(SD)	40.1 (11.5)
Mean pre‐pregnancy weight (*n* = 35), kg (SD)	89.8 (24.5)

GDM was diagnosed at 25^+3^ (± 7^+1^) weeks gestation. A 75 g OGTT was performed in 3/36 (8.3%), of which 1/36 participants experienced hypoglycaemia at a glucose level of 3.2 mmol/L while the remainder were diagnosed following a week of capillary glucose monitoring. In the overall cohort, HbA1c was 5.1% (± 0.3%, *n* = 10) in first trimester, 4.9% (± 0.3%, *n* = 12) in second trimester and 5.0% (± 0.3%, *n* = 15) in third trimester. An Endocrinologist reviewed 33/36 (91.6%) of women following diagnosis with first contact occurring at 26^+3^ (± 7^+1^). Insulin therapy was commenced in 16/36 (44.4%) at mean gestation 28^+3^ (± 5^+2^) with a total daily dose of 20.3 (± 12.7)iu in the late third trimester. Almost half of the women in our cohort commenced insulin, compared to the National rate for women with GDM of 31.7% [21]. Recurrent hypoglycaemia occurred in 10/36 women (27.8%), with one experiencing fasting hypoglycaemia, three post‐prandially, three both fasting and post‐prandially and three for which the timing was not specified. Six women had hypoglycaemic episodes that were documented as limiting insulin management or titration. Of these six women, three had previously experienced hypoglycaemia after metabolic surgery, prior to conception Of the subset where HbA1c was available there was a trend towards higher HbA1c for women requiring insulin compared to diet controlled (5.1%, *n* = 4/16 vs. 4.9%, *n* = 8/20 in second trimester and 5.1%, *n* = 5/16 vs. 5.0%, *n* = 10/20 in third trimester; *p* > 0.05).

Dietetic review occurred in 34/36 (94.4%) of women at 26^+5^ (± 6^+2^), with a subsequent review in 13/36 (36.1%). Carbohydrate intake was assessed according to the American Diabetic Association recommendation of minimum of 175 g carbohydrate per day for GDM [22]; 10/34 (29.4%) women had adequate intake, the majority were considered by the antenatal dietitian to be inconsistent (20.6%), inadequate (32.4%), or excessive (2.9%), with the remainder undocumented (20.6%). Gestational weight gain was recorded in 5/36 (13.8%). Only 1/35 (2.7%) participants were comprehensively screened for nutritional adequacy according to our local protocol (Table 2a, 2b). The most common vitamin deficiencies identified were iron deficiency (33/36, 91.7%), zinc deficiency (13/27, 48.1%), B12 deficiency (17/36, 47.2%) and vitamin D deficiency (11/36, 30.6%). Most women (26/36, 72.2%) were deficient in more than one micronutrient with a median of two micronutrient deficiencies per woman.

**TABLE 2a:** Micronutrient deficiencies screened during pregnancy.

Micronutrient	*n* deficient/*n* screened (%)
Zinc	13/27 (48.1)
Copper	0/25 (0.0)
Selenium	1/26 (3.8)
INR (as surrogate for Vitamin K)	0/23 (0.0)
B1	0/22 (0.0)

**TABLE 2b:** Micronutrient deficiencies screened each trimester.

Micronutrient	Trimester 1 *n* deficient/*n* screened (%)	Trimester 2 *n* deficient/*n* screened (%)	Trimester 3 *n* deficient/*n* screened (%)	Total *n* deficient/*n* screened (%)
Iron	8/24 (33.3)	12/17 (70.6)	29/32 (90.6)	33/36 (91.7)
B12	5/19 (26.3)	5/19 (26.3)	10/30 (33.3.)	17/36 (47.2)
Vitamin D	5/20 (25.0)	3/17 (17.6)	6/25 (24.0)	11/36 (30.6)
Folate	0/15 (0.0)	0/12 (0.0)	0/20 (0.0)	0/29 (0.0)

Gestational hypertension occurred in 1/36 (2.8%) women and pre‐eclampsia in 2/36 (5.6%) women. Gestational age at delivery was 38^+1^ (± 1^+5^) with 1/36 (2.8%) women birthing prematurely under 37 weeks gestation. Delivery was by Caesarean section for 17/36 (47.2%) women, of which 6/17 (35.3%) were unplanned surgeries. Neonatal birth weight was 3270.1 (± 349.9) g. The composite of adverse neonatal outcomes occurred in 22/36 (61.1%) of births (Table 3). Neonatal hypoglycaemia occurred in 8/36 (22.2%) of neonates; 1/36 (2.8%) were small‐for‐gestational age, and 2/36 (5.6%) were large‐for‐gestational age. Resuscitation was required for 7/36 (19.4%) of neonates and 10/36 (27.8%) of neonates were admitted to NICU. A higher neonatal birthweight was observed in women requiring insulin (3418 g vs. 3153 g, *p* = 0.022). Women with recurrent hypoglycaemia (10/36, 27.8%) had trend towards birthing smaller babies (3104 g vs. 3334 g, *p* = 0.077) compared to women without hypoglycaemia.

**TABLE 3:** Maternal and neonatal outcomes of women with GDM after metabolic surgery (*n* = 36) compared to the available Australian Institute of Health & Wellbeing data for women with GDM and the Australian Diabetes in Pregnancy Society 2021 benchmarking data.

Outcome	*n* (%)	Australian GDM data, % [7]	ADIPS benchmarking data, % [21]
Maternal outcomes			
Gestational hypertension	1 (2.8)	5.2	—
Pre‐eclampsia	2 (5.6)	3.0	—
Caesarean section	17 (47.2)	40.4	20.3
Neonatal outcomes			
Prematurity (< 37/40)	1 (2.8)	10.9	9.5
Shoulder dystocia	1 (2.8)	—	2.8
Resuscitation	7 (19.4)	20.6	—
Respiratory distress	11 (30.6)	—	—
NICU^a^ admission	10 (27.8)	26.5	21.2
Neonatal hypoglycaemia (< 2.8 mmol/L)	8 (22.2)	—	16.9
Jaundice	5 (13.9)	—	4.9
Large for gestational age at birth (> 90th centile)	2 (5.6)	—	13.1
Small for gestational age at birth (< 10th centile)	1 (2.8)	—	9.3
Composite outcomes^b^	22 (61.1)	—	—

^a^ Neonatal intensive care unit.

^b^ Neonatal composite outcomes defined as any occurrence of neonatal hypoglycaemia (< 2.8 mmol/L), small‐for‐gestational age at birth (< 10th centile), large‐for‐gestational age at birth (> 90th centile), jaundice, neonatal intensive care admission, respiratory distress, resuscitation or shoulder dystocia.

**FIGURE 1:**
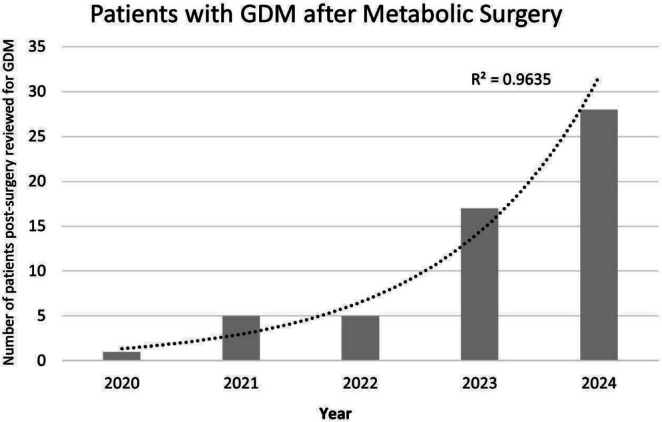
Increasing incidence of GDM after metabolic surgery.

The antenatal endocrine clinic has provided services to an increasing number of women with GDM after metabolic surgery each year (Figure 1). This contrasts to the numbers of women with pre‐existing diabetes, which has remained relatively stable in our centre at approximately 50 per year. In 2023, for comparison, there were 26 women with Type 1 diabetes, 22 women with Type 2 diabetes, and 17 women with GDM after metabolic surgery. Our data suggests that at the current rate of increase, patients with GDM after metabolic surgery will outnumber pregnant women with pre‐existing Type 1 or Type 2 diabetes in our centre by 2025.

## 4 Discussion

Gestational diabetes mellitus after metabolic surgery is an increasingly encountered issue in antenatal services. Nutritional deficiencies are common, and carbohydrate intake is often inadequate. Carbohydrate intake is critical to fetal growth [22]. Given that a minority of women in our cohort had adequate carbohydrate intake at the time of initial dietitian review, monitoring of fasting urinary ketones may be a useful adjunct. In keeping with previous studies [14, 15], we demonstrated that where screening is performed, many women have micronutrient deficiencies in pregnancy following metabolic surgery. The gestation age at time of first review with a dietitian was later in pregnancy after organogenesis had occurred, highlighting the importance of comprehensive early screening and appropriate supplementation. To reduce the risk of micronutrient deficiency complicating pregnancy, dietitian review should be incorporated into routine preconception and early pregnancy management [23].

There was a high rate of composite adverse neonatal outcome suggesting that these pregnancies may be higher risk. However, the maternofetal outcomes were similar to national population data for women with GDM without metabolic surgery [7, 21]. The established risk of small‐for‐gestational‐age babies following metabolic surgery [5] was not reflected in this cohort of women with GDM with only one SGA baby, which could reflect variation in diagnostic criteria, glycaemic targets, or diabetes management. Furthermore, women with hypoglycaemia had smaller babies whereas larger babies were born to women requiring insulin which may be a consequence of maternal hyperglycaemia and fetal hyperinsulinaemia contributing to fetal growth.

Further research is warranted to develop guidelines for GDM after metabolic surgery. There is currently no consensus on the diagnostic approach or criteria and local hospitals have developed their own protocols. Early pregnancy screening at around 6 weeks allows identification of pre‐existing dysglycaemia if fasting glucose ≥ 5.1 mmol/L and/or HbA1c 5.9% [24]. There is inconsistent support for later screening with an OGTT, due to the increased risk of hypoglycaemia in this group [25]. It should be noted that if an OGTT has been performed, it may be unadvisable to base diagnosis solely on the 1‐h glucose threshold in this context due to rapid glycaemic excursion [16]. The American College of Obstetricians and Gynaecologists (ACOG) and the Royal Australian and New Zealand College of Obstetricians and Gynaecologists (RANZCOG) advise capillary blood glucose monitoring for approximately 1 week from 24 to 28 weeks with measurement of fasting and postprandial glucose levels and additional measurements if there are symptomatic hyperglycaemic or hypoglycaemic events [4]. Recommended diagnostic thresholds using OGTT differ given the non‐uniform criteria for GDM diagnosis internationally. One approach suggests thresholds of fasting plasma glucose ≥ 5.1 mmol/L, or 2‐h postprandial ≥ 8.5 mmol/L [24] or HbA1c ≥ 6.5% [15]. If late dumping syndrome (a hyperinsulinaemic response to ingested carbohydrate producing postprandial reactive hypoglycaemia) is suspected, additional postprandial measurements beyond the 2‐h measurement are necessary and recommended [15]. Alternatively, it has been proposed that a GDM diagnosis may be made at 24–28 weeks if 20% of all self‐monitored capillary glucose levels exceed target before meals ≥ 5.3 mmol/L, 1‐h post meal ≥ 7.8 mmol/L, 2‐h postprandial ≥ 6.7 mmol/L [24] or perhaps > 6.4 mmol/L [4]. However, as many women remain high‐risk for GDM after surgery, screening should be considered earlier in gestation with one approach suggesting commencement of monitoring from 14 to 16 weeks, continued through pregnancy [4], whilst acknowledging the additional demand this places on women and healthcare resources. Critically, guidelines for the optimal glucose targets for women with GDM after metabolic surgery remain undefined, although avoidance of hypoglycaemia has been identified as a priority. Indeed, it has been proposed standard glucose targets should be modified to avoid hypoglycaemia and the consequent negative effects on fetal growth [26].

Continuous glucose monitoring (CGM) is now widely used in patients with Type 1 diabetes mellitus and less commonly in people with GDM. Use of CGM in patients with GDM after metabolic surgery reduces the risk of hypoglycaemic events by detecting hyperglycaemic peaks and imminent hypoglycaemia and is particularly useful for patients with hypoglycaemia unawareness: a lack of hypoglycaemic symptoms consequential to repeated hypoglycaemic exposure [27]. Self‐initiated lifestyle modifications in response to real‐time glucose levels from CGM can improve glycaemic control, reduce glycaemic variability and improve pregnancy outcomes, with one study demonstrating reduction in fetal macrosomia from 20% to 4% in a general population of women with GDM [28]. CGM is safe and accurate in pregnant women [29]. Further studies are necessary to evaluate CGM accuracy and to clarify the optimal diagnostic GDM thresholds and targets in women after metabolic surgery.

To the best of our knowledge, this is the first cohort reporting antenatal, maternal and neonatal outcomes in women with GDM after metabolic surgery. Additionally, the vast majority of this cohort had a history of sleeve gastrectomy for which there is minimal pregnancy data available in the literature compared to Roux‐en‐Y gastric bypass despite sleeve gastrectomy being performed much more commonly [12]. A recent Australian data‐linkage study by Eccles‐Smith et al. [30] provided some local outcome data in a population that largely underwent sleeve gastrectomy, although this did not specifically review the maternal and neonatal outcomes of women with GDM. Interestingly, this did not demonstrate an increased risk of micronutrient deficiencies as seen in our study. Given the retrospective nature of our study there was an absence of data regarding attendance of preconception counselling or supplementation of micronutrient deficiencies. A further limitation was the incomplete data for the parameters of gestational weight gain and HbA1c. Capillary glucose levels were not included in the standard audit template or documented in the electronic medical record and therefore glucose variability was not calculable. The research team is embarking on a prospective study including these important variables; collaborations with other units would enable generation of larger data sets to address the critical unknown criteria for GDM diagnosis and subsequent glucose targets during pregnancy. The use of CGM for early diagnosis of GDM is an exciting area of current research [31, 32] and expansion of study cohorts to include women with GDM after metabolic surgery is imperative.

## Conflicts of Interest

The authors declare no conflicts of interest.
